# EGF Receptor-Dependent Mechanism May be Involved in the Tamm–Horsfall Glycoprotein-Enhanced PMN Phagocytosis via Activating Rho Family and MAPK Signaling Pathway

**DOI:** 10.3390/molecules19011328

**Published:** 2014-01-21

**Authors:** Ko-Jen Li, Sue-Cien Siao, Cheng-Han Wu, Chieh-Yu Shen, Tsai-Hung Wu, Chang-Youh Tsai, Song-Chou Hsieh, Chia-Li Yu

**Affiliations:** 1 Institute of Clinical Medicine, National Yang-Ming University College of Medicine, Taipei 11221, Taiwan; E-Mail: dtmed170@yahoo.com.tw; 2 Institute of Molecular Medicine, National Taiwan University College of Medicine, Taipei 10002, Taiwan; E-Mail: syuecian@gmail.com; 3 Institute of Clinical Medicine, National Taiwan University College of Medicine, Taipei 10002, Taiwan; E-Mails: chenghanwu@ntu.edu.tw (C.-H.W.); tsichhl@gmail.com (C.-Y.S.); 4 Section of Nephrology, Taipei Veterans General Hospital, Taipei 11221, Taiwan; E-Mail: wuth@vghtpe.gov.tw; 5 Section of Allergy, Immunology & Rheumatology, Taipei Veterans General Hospital, Taipei 11221, Taiwan; E-Mail: cytsai@vghtpe.gov.tw; 6 Department of Internal Medicine, National Taiwan University Hospital and National Taiwan University College of Medicine, Taipei 10002, Taiwan; E-Mail: hsiehsc@ntu.edu.tw

**Keywords:** Tamm-Horsfall glycoprotein, phagocytosis, EGF-like domains, Rho family, MAP kinase

## Abstract

Our previous studies showed that urinary Tamm–Horsfall glycoprotein (THP) potently enhanced polymorphonuclear neutrophil (PMN) phagocytosis. However, the domain structure(s), signaling pathway and the intracellular events responsible for THP-enhanced PMN phagocytosis remain to be elucidated. THP was purified from normal human urine. The human promyelocytic leukemia cell line HL-60 was induced to differentiate into PMNs by all-trans retinoid acid. Pretreatment with different MAPK and PI3K inhibitors was used to delineate signaling pathways in THP-enhanced PMN phagocytosis. Phosphorylation of molecules responsible for PMN phagocytosis induced by bacterial lipopolysaccharide (LPS), THP, or human recombinant epidermal growth factor (EGF) was evaluated by western blot. A p38 MAPK inhibitor, SB203580, effectively inhibited both spontaneous and LPS- and THP-induced PMN phagocytosis. Both THP and LPS enhanced the expression of the Rho family proteins Cdc42 and Rac that may lead to F-actin re-arrangement. Further studies suggested that THP and EGF enhance PMN and differentiated HL-60 cell phagocytosis in a similar pattern. Furthermore, the EGF receptor inhibitor GW2974 significantly suppressed THP- and EGF-enhanced PMN phagocytosis and p38 and ERK1/2 phosphorylation in differentiated HL-60 cells. We conclude that EGF receptor-dependent signaling may be involved in THP-enhanced PMN phagocytosis by activating Rho family and MAP kinase.

## 1. Introduction

Urinary Tamm–Horsfall glycoprotein (THP) or uromodulin is an 80–90 kDa GPI-anchored protein synthesized by renal tubular cells in the thick ascending limb of Henle’s loop [1]. The daily urinary THP excretion by normal individuals is about 50–200 mg [2]. THP plays an important role in protecting the kidneys and urinary tract from pathogenic microbe invasion [3]. Some authors have reported that THP-deficient mice are highly susceptible to severe urinary system infections [4]. Because THP is confined to the urinary system, anti-THP antibodies can be easily elicited in patients with chronic pyelonephritis once THP begins to leak into the renal interstitium [5,6]. Genetic mutations in the THP molecule may result in different renal outcomes, ranging from renal salt wasting and hyperuricemia/gout to renal failure [7,8,9].

The THP molecule contains approximately 25%–35% carbohydrate side chains with abundant sialic acid [10]. The protein core of the THP molecule is composed of a N-terminal signal peptide, three epidermal growth factor (EGF)-like domains, eight conserved cysteine residue domains (D8C), and a zona pellucida domain (ZP) [10,11]. It is conceivable that the major glycomoiety of THP is N-linked glycans [12]. The high-mannose glycans are carried by Asn_251_, which mediates the interaction with type 1 fimbriated *Escherichia coli* in order to prevent *E. coli* from attaching to urinary epithelial cells [13,14]. The minor O-linked carbohydrate side chains in THP exhibit binding affinities to different protein molecules [15]. These THP-binding proteins include IL-1 [16], TNF-α [17], immunoglobulin light chains [18], and the complement components 1 and 1q [19]. Many research groups, including our own, have shown that THP enhances the phagocytic activity of polymorphonuclear neutrophils (PMNs) [20,21,22]. Saemann *et al.* [23] further found that THP, as a regulatory factor, could activate myeloid dendritic cells becoming a full mature dendritic phenotype via a toll-like receptor-4-dependent mechanism. Our recent study demonstrated that THP binds to lactoferrin and cathepsin G, which are expressed on the PMN surface, to enhance PMN phagocytosis [24]. Contradictory to the results of Easton *et al.* [15], we noted that the intact protein-core structure, rather than the carbohydrate side chains, was responsible for THP-enhanced PMN phagocytosis [25]. However, the domain structure(s), signaling pathways and intracellular events responsible for THP-enhanced PMN phagocytosis remain unclear. In the present study, functional assessments and THP-transduced signaling pathways were investigated to explore the molecular basis of THP-enhanced PMN phagocytosis. 

## 2. Results and Discussion

### 2.1. THP Enhances PMN Phagocytosis

**Figure 1:**
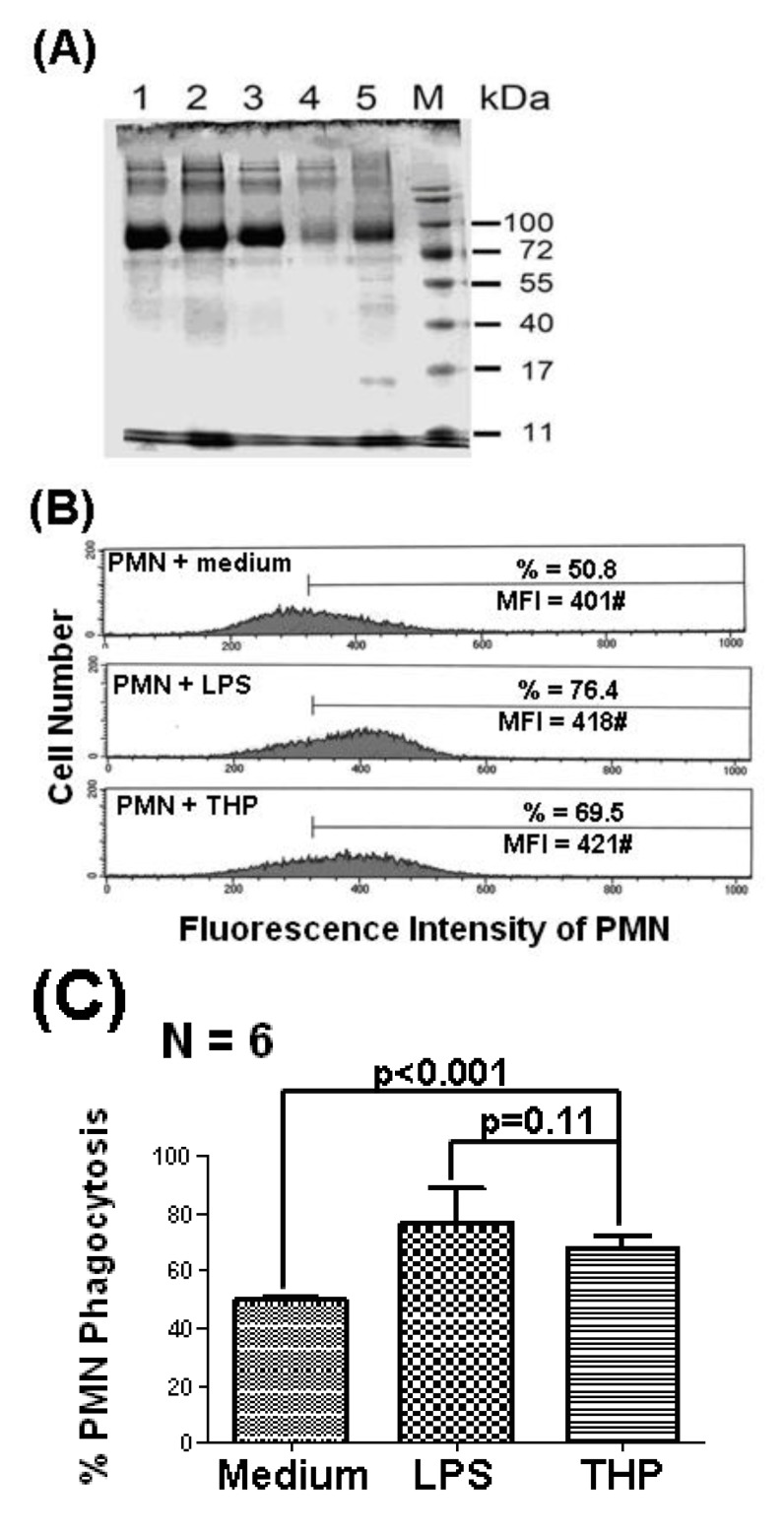
THP (10 μg/mL) purified from normal human urine enhances PMN phagocytosis, as detected by flow cytometry. (**A**) The molecular weight (80–90 kDa) of five different THP specimens was estimated by 10% SDS-PAGE. Some large THP aggregates were demonstrated in the higher molecular weight bands. (**B**) A typical case showing THP-enhanced PMN phagocytosis. LPS (20 ng/mL)-activated PMN phagocytosis was used as the positive control. (**C**) PMN phagocytosis resulting from spontaneous (medium), LPS, and THP activation was compared.

Our previous reports demonstrated that THP potently enhanced PMN phagocytosis [22,24,25]. We confirmed again that the 80–90 kDa THP molecule, purified from five different normal human urine samples (Figure 1A), significantly enhanced PMN phagocytosis, as shown in Figure 1C. A representative case is shown in Figure 1B. The higher molecular weight molecules in each lane were aggregates of THP. The identification of THP was confirmed by staining with anti-uromucoid antibodies (data not shown) as shown in our previous report [22].

### 2.2. The p38 MAP Kinase Signal Pathway is Involved in Spontaneous, Bacterial Lipopolysaccharide- Enhanced, and THP-Enhanced PMN Phagocytosis

**Figure 2:**
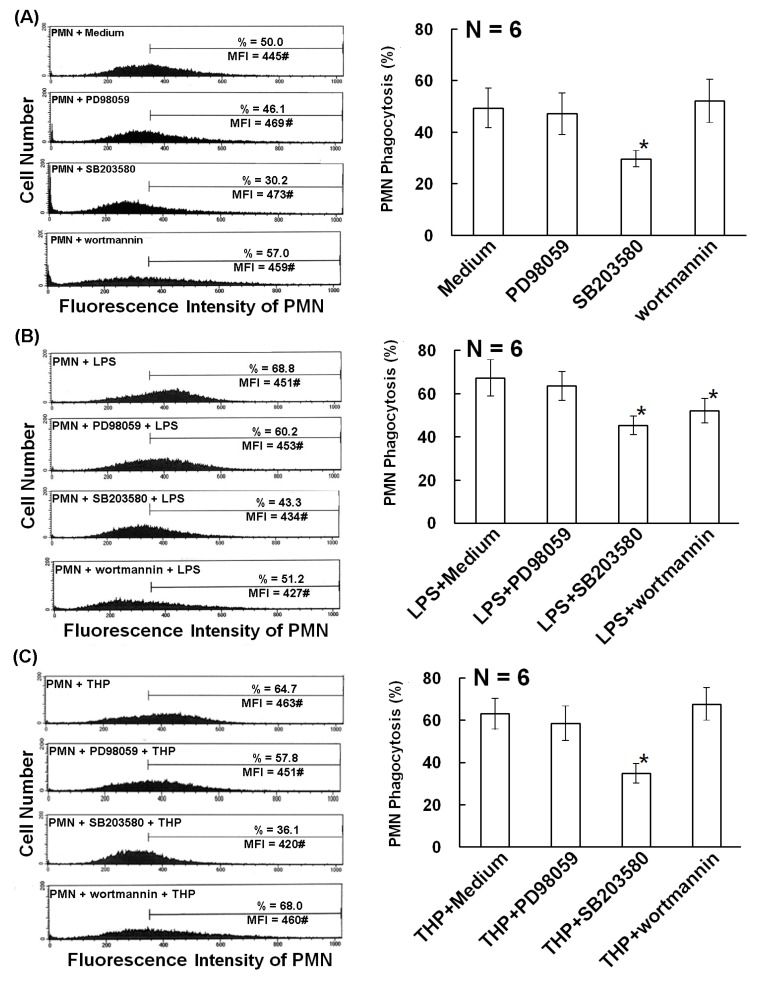
Effects of different protein kinase inhibitors on spontaneous, LPS (20 ng/mL)-, and THP (10 μg/mL)-induced phagocytosis. (**A**)–(**C**): The left panel shows a representative case and the right panel shows statistical assessment. (**A**) Spontaneous PMN phagocytosis was detected after pretreatment with different protein kinase inhibitors including PD98059 (50 μM, a non-competitive inhibitor of MAPK kinase 1), SB203580 (1 μM, a p38 inhibitor), or wortmannin (0.1 μM, a specific covalent inhibitor of PI3K). (**B**) LPS-stimulated PMN phagocytosis was detected after pretreatment with different protein kinase inhibitors as demonstrated in (**A**). (**C**) THP-stimulated PMN phagocytosis was measured after pretreatment with different protein kinase inhibitors as above. * denotes *p* ≤ 0.05 compared to medium control.

For elucidating the molecular basis of THP-enhanced PMN phagocytosis, our next step was to identify the signal pathways transduced by THP. Normal human PMNs were pretreated with different protein kinase inhibitors including PD98059 (a non-competitive inhibitor of MKK1), SB203580 (a p38 MAPK inhibitor), and wortmannin (a specific covalent inhibitor of phosphoinositide 3 kinase, PI3K) for 20 min. Spontaneous, bacterial lipopolysaccharide (LPS)- and THP-stimulated PMN phagocytosis was measured by flow cytometry. We found that the p38 MAPK inhibitor SB203580 significantly suppressed spontaneous (Figure 2A), LPS-induced (Figure 2B), and THP-induced (Figure 2C) PMN phagocytosis. In addition, wortmannin also suppressed LPS-induced phagocytosis (Figure 2B). These results suggest that the p38 MAPK signaling pathway is involved in both spontaneous and PMN activator-enhanced PMN phagocytosis. In addition to p38 MAPK signaling, PI3K signaling is also involved in LPS-enhanced phagocytosis.

**Figure 3:**
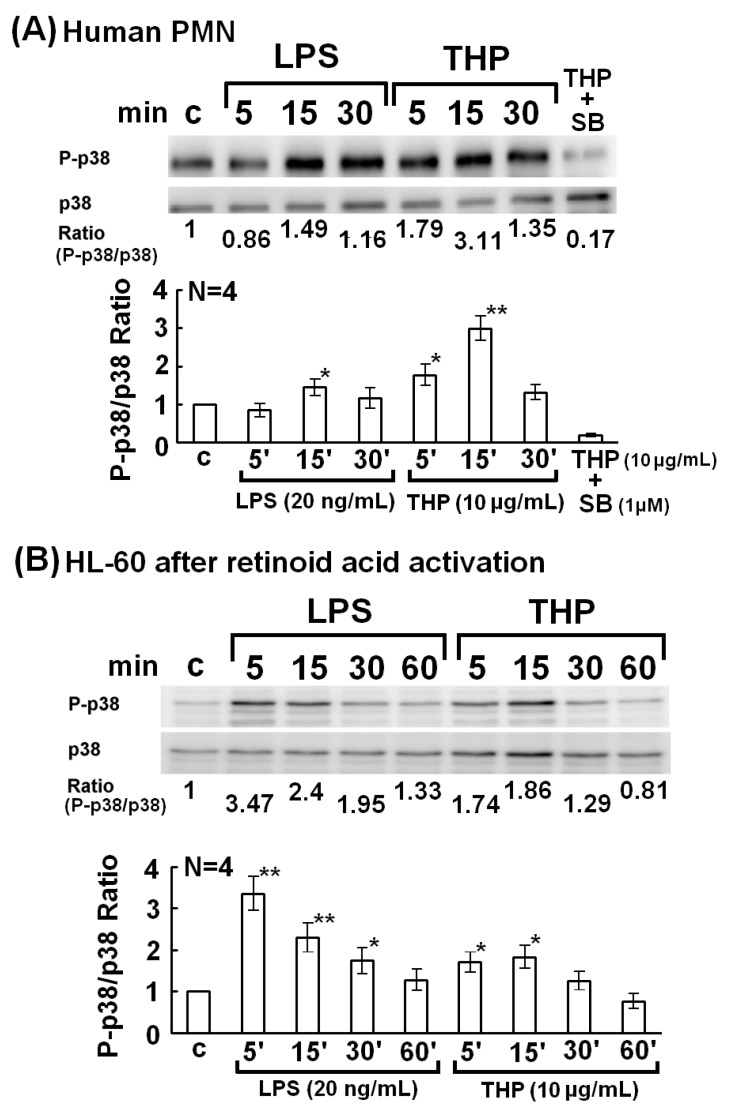
Kinetic expression of p38 MAPK phosphorylation by THP (10 μg/mL) and LPS (20 ng/mL) stimulation from 5–60 min in normal human PMNs (**A**) and all-trans retinoic acid (2 μM)-activated human promyelocytic leukemia HL-60 cells (**B**). The upper panel shows a representative case and the lower panel shows the statistical assessment in both (**A**) and (**B**). * denotes *p* ≤ 0.05 and ** denotes *p* ≤ 0.01 compared to control.

To further confirm the importance of p38 MAPK in PMN phagocytosis, normal human PMNs (Figure 3A) or all-trans retinoid acid-activated HL-60 cells (Figure 3B) were stimulated with LPS or THP for 5–30 min. The p38 MAPK phosphorylation was detected by western blot (Figure 3). Kinetic studies revealed that p38 MAPK phosphorylation in THP-stimulated human PMNs was not only faster (5 min *vs.* 15 min) but also higher (p-p38/p38 ratio = 3.28 ± 0.42 *vs.* 1.71 ± 0.21) than in LPS-activated PMNs (Figure 3A). In addition, the p38 MAPK inhibitor SB203580 (1 μM) remarkably suppressed THP-induced phospho-p38 expression (THP + SB in Figure 3A). Conversely, LPS-induced p38 phosphorylation in differentiated HL-60 cells was faster (5 min *vs.* 15 min) and higher (p-p38/p38 ratio = 3.54 ± 0.62 *vs.* 1.64 ± 0.28) than THP activation (Figure 3B). These results indicate that LPS and THP induce different degrees and kinetic changes of p38 MAPK phosphorylation depending on the origin of the phagocytes.

**Figure 4:**
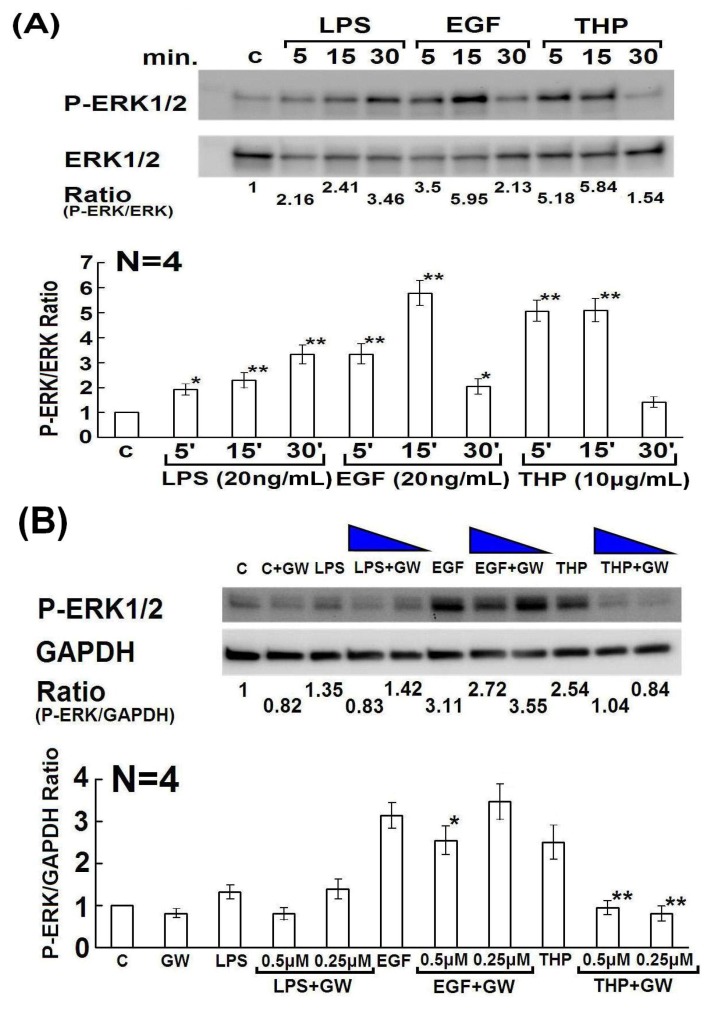
In panels (**A**) and (**B**), the upper panels are representative cases and lower panels show the statistical assessment. (**A**) Kinetic induction of phospho-ERK1/2 in HL-60 cells by LPS, EGF, and THP from 5 to 30 min. (**B**) Inhibitory effects of GW2974 (0.25 μM and 0.5 μM) on LPS-, EGF-, and THP-induced phospho-ERK1/2 in differentiated HL-60 cells. * denotes *p* ≤ 0.05 compared to EGF and ** denotes *p* ≤ 0.01 compared to THP.

In our previous report, we found that THP-induced p38 MAPK phosphorylation was decreased when human IgGs were used as molecular-weight control [24]. In the present study, we used 10% FBS-RPMI as the negative control and bacterial LPS as the positive control for THP. We found that THP not only enhanced the expression of p38 but also that of ERK1/2 MAPKs (Figure 4). It is conceivable that the MAPK signaling pathway is crucial for cells to adapt to different environmental stresses such as nutrient concentration, UV irradiation, growth factors, and cytokines [26]. Our results suggest that stimulation of PMN by THP or LPS activates the MAPK signaling pathway in the cells in response to environmental stresses.

### 2.3. Involvement of Rho Family GTPase Molecules in THP-Enhanced PMN Phagocytosis

**Figure 5:**
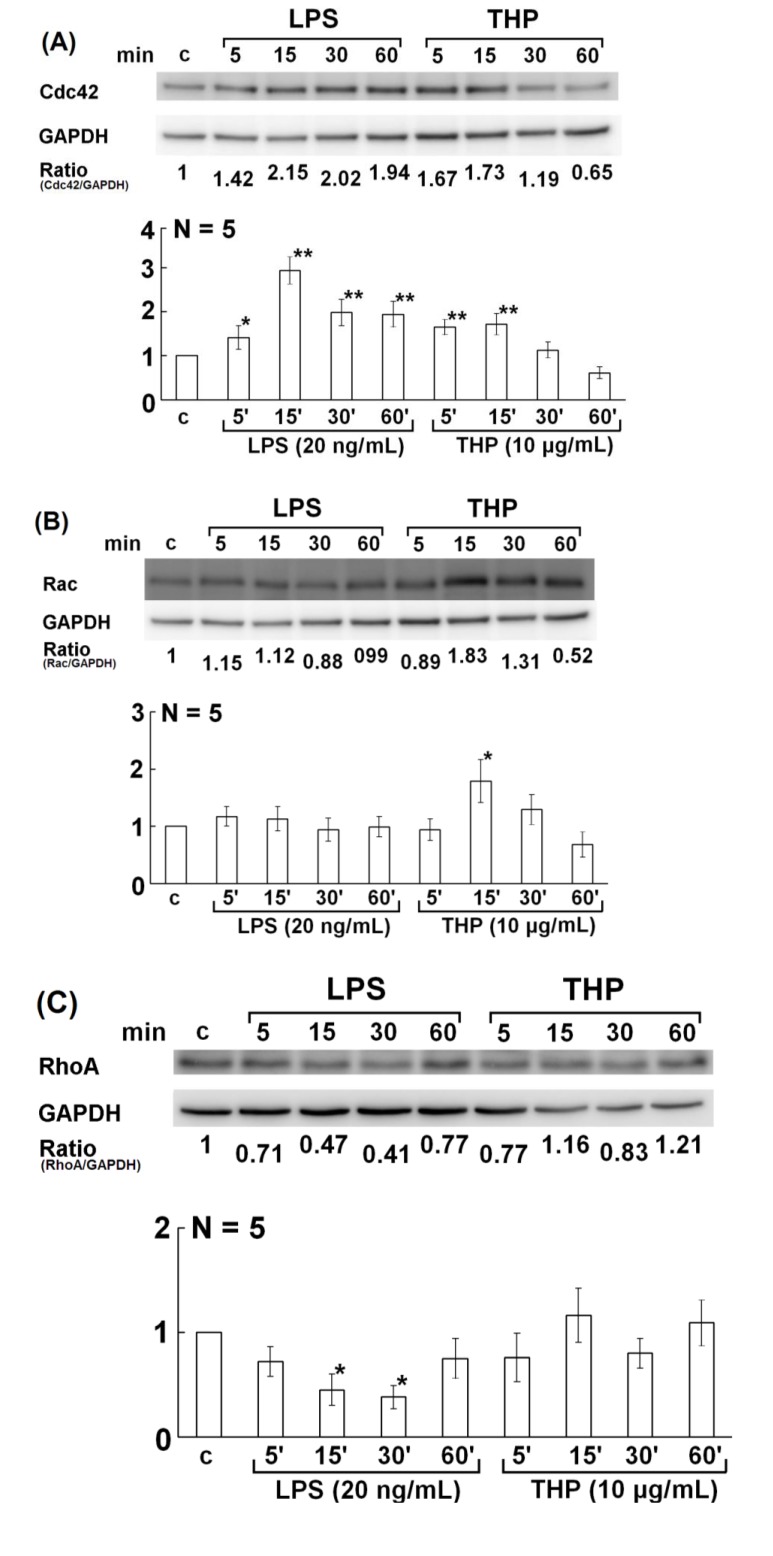
Kinetic expression of the Rho family small GTPase molecules Cdc42, Rac, and RhoA in HL-60 cells after stimulation with LPS (20 ng/mL) or THP (10 μg/mL) for 5–60 min and then detected by western blot. In panels (**A**–**C**), the upper panel is a representative case and the lower panel shows the statistical assessment. (**A**) Cdc42, (B) Rac, and (**C**) RhoA. * denotes *p* ≤ 0.05 and ** denotes *p* ≤ 0.01 compared to control.

Many authors reported that the Rho family of small GTPase molecules, including Cdc42, Rac1, and RhoA, link membrane receptors to the actin cytoskeleton and may control a variety of cell signaling functions including phagocytosis [27,28,29]. To determine which of these small GTPase molecules mediates THP- or LPS-enhanced PMN phagocytosis, we looked for the 3 Rho family molecules in differentiated HL-60 cells. We found that Cdc42 expression was enhanced by THP and LPS stimulation (Figure 5A). In contrast, Rac levels were increased only in THP-activated HL-60 cells (Figure 5B). However, RhoA expression decreased on LPS stimulation and showed no change upon THP stimulation (Figure 5C). These results suggest that THP-enhance PMN phagocytosis depends on Cdc42 and Rac, which is somewhat different from LPS.

It is believed that immunoglobulin-opsonized particles are recognized by surface-expressed immunoglobulin Fc receptors (FcR) and that the Rho family members Cdc42 and Rac then initiate local cytoskeleton rearrangement [30]. We found that the expression of Cdc42 and Rac molecules was increased in THP-stimulated PMNs and Cdc42 was increased in LPS-activated PMNs (Figure 5A,B). It appears that Cdc42 and Rac are more important than RhoA in eliciting F-actin remodeling. Recently, a study showed that Cdc42 and PI3K interact, resulting in actin polymerization during FcR-mediated PMN phagocytosis [30]. Furthermore, p38 and ERK1/2 MAPK signaling pathways are located downstream of GTPase-mediated F-actin rearrangement. 

Indeed, we noted that wortmannin (a PI3K inhibitor) significantly reduced LPS-induced phagocytosis from 65.4% ± 4.17% to 48.37% ± 4.52%, as shown in Figure 2B. However, wortmannin did not reduce spontaneous or THP-induced PMN phagocytosis (Figure 2A,C). These results suggest that different PMN stimulators activate different signaling pathways in PMNs. We hypothesized that LPS mainly activates the Cdc42, p38 and ERK1/2 MAPK, and PI3K signaling pathways. In contrast, THP and EGF mainly activate the Cdc42, Rac, and p38 and ERK1/2 signaling pathways. 

### 2.4. Determination of Domain Structure(s) in the THP Protein Core Responsible for Enhancing PMN Phagocytosis and Their Signaling Pathways

**Figure 6:**
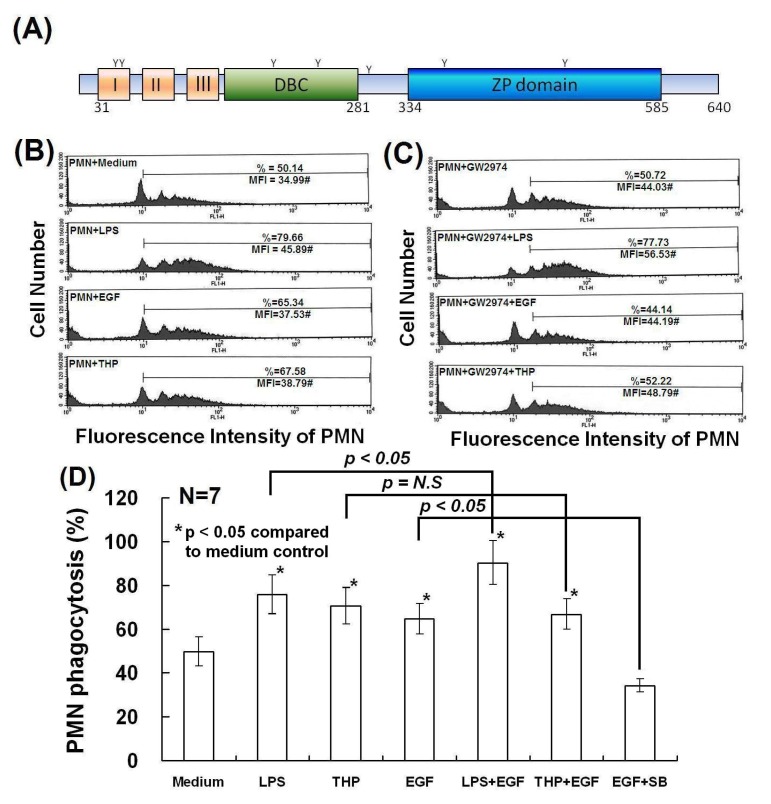
A schematic domain structure of the THP protein core and the effect of recombinant human EGF (20 ng/mL) and EGFR inhibitor GW2974 (0.25 μM) on PMN phagocytosis. (**A**) Domain structure of THP’s protein core. Black box: leader peptide; orange boxes: EGF-like domains; green box: D8C domain; blue box: ZP domain. The 7 N-glycosylation sites are marked as Y. (**B**) PMNs were activated by LPS, EGF, or THP for 30 min, and then PMN phagocytosis was detected by flow cytometry. (**C**) PMNs were pretreated with GW2974 (0.25 μM) for 20 min followed by stimulation with LPS, EGF, or THP for 30 min. PMN phagocytosis was detected by flow cytometry. (**D**) Comparison of PMN phagocytosis among different treated groups. * denotes *p* ≤ 0.05 compared to medium control. It is worthy to note that (LPS+EGF)-induced PMN phagocytosis is higher than LPS alone (*p* < 0.05) whereas (THP+EGF)-induced phagocytosis is not different from THP alone. A p38 MAPK inhibitor, SB203580, potently suppressed EGF-enhanced PMN phagocytosis.

We previously demonstrated that the protein core, rather than the carbohydrate side chains, in the THP molecule was responsible for THP-enhanced PMN phagocytosis [25]. In the present study, we intended to explore the domain structure(s) of THP that influences this activity. As shown in Figure 6A, three EGF-like domains are present after the leader peptide in the THP protein core. Schmidt *et al.* [31] demonstrated that the EGF-like domain 7, secreted by endothelial cells containing two EGF-like domains, could modulate Notch signaling for neural stem cell renewal [31]. The authors further demonstrated that EGF mediates a number of biological roles in phagocyte biology, including cytoskeletal remodeling, cell adhesion, and phagocytosis [32,33,34]. Based on these observations, we hypothesized that the three EGF-like domains in the THP protein core may be important for THP-enhanced phagocytosis. As shown in Figure 6B, the degree of PMN phagocytosis induced by EGF was comparable to that induced by THP.

The specific EGFR inhibitor GW2974 effectively suppressed PMN phagocytosis induced by both EGF and THP, but not LPS (Figure 6C). In addition, treatment of PMN with THP + EGF did not increase phagocytosis (*p* = N.S.), whereas treatment with LPS + EGF additively enhanced PMN phagocytosis (*p* < 0.05) (Figure 6D). In addition, the p38 MAPK inhibitor SB203580 suppressed EGF-enhanced (*p* < 0.05) (Figure 6D) as well as THP-enhanced PMN phagocytosis (Figure 2C). These results indicate that THP enhances PMN phagocytosis via the EGF receptor-dependent signaling after THP’s protein binding. Kansas *et al.* [32] found that the EGF-like domain of P-selectin plays an important role in ligand recognition and cell adhesion. These results suggest that EGF-like domains in the THP protein core are potentially involved in protein-protein or ligand-receptor interactions of THP with other molecules. However, more investigations are required to confirm this hypothesis.

Lastly, we tried to demonstrate whether EGF-EGFR activation also transduces signals via ERK1/2 and MAPK phosphorylation as reported by other authors [35,36]. We compared the signaling pathways elicited by human recombinant EGF and THP in the present study. The phosphorylation of ERK1/2 induced by THP and EGF exhibited the same kinetics and strength as shown in Figure 4A. The phosphorylation of ERK1/2 was also suppressed by the EGFR inhibitor GW2974 (Figure 4B]. Our previous reports demonstrated that THP-enhanced PMN phagocytosis is mediated by the binding of the THP protein core to lactoferrin and cathepsin G expressed on the PMN surface [22,24,25]. Based on these findings, we propose that the THP protein core may also stimulate EGF receptors in addition to lactoferrin and cathepsin G to transduce the EGFR signals and enhance PMN phagocytosis [24]. Whether the 3 EGF-like domains in THP protein core directly involving in EGFR signaling needs further investigations by THP genetic manipulation including deleting or mutating the nucleotide sequences of the EGF-like domains.

## 3. Experimental

### 3.1. Reagents and Antibodies

Fluoresbrite carboxylate microspheres were purchased from Polysciences, Inc. (Worrington, PA, USA). LPS, *E. coli* serotype 026:B6), 4',6-diamidino-2-phenylindole (DAPI), all-trans retinoid acid, GW2974 (an EGFR inhibitor), and wortmannin (a specific inhibitor of PI3K) were obtained from the Sigma-Aldrich Immunochemical Company (St. Louis, MO, USA). Fluorescent phalloidin was obtained from Molecular Probes (Eugene, OR, USA). PD98059 (a non-competitive inhibitor of MAPK kinase 1, MKK1) and SB203580 (a p38 MAPK inhibitor) were purchased from Calbiochem, Inc. (San Diego, CA, USA). Recombinant human EGF was obtained from R&D Systems, Inc. (Minneapolis, MN, USA). Mouse monoclonal antibodies against human p38 MAPK, phospho-p38 MAPK, ERK1/2, phospho-ERK1/2, Cdc42, Rac, and RhoA were purchased from Cell Signaling Technology, Inc. (Danvers, MA, USA). HRP-conjugated goat anti-mouse IgGs were obtained from Immunoresearch Laboratories (West Grove, PA, USA).

### 3.2. Purification of THP from Pooled Normal Human Urine

Twenty-four hour urine was collected in a clean bottle from normal individuals and kept at 4 °C. The purification of THP followed the method reported by Hunt *et al.* [37]. Briefly, the urine sample was made up to 0.58 M NaCl with continuous stirring for 30 min at 4 °C, followed by centrifugation at 2000 ×*g* for 20 min to precipitate THP. The precipitates were dissolved in alkaline distilled water (pH 9.0) adjusted with 1N NaOH, and then precipitated in 0.58 M NaCl. The precipitation-dissolution cycle was repeated at least three times. The purified THP solution was dialyzed in a cellulose membrane tube (spectrum, MWCO: 8000) against distilled water at 4 °C overnight. The obtained THP in distilled water was finally lyophilized and stored at −20 °C until use. For experiments, purified THP was dissolved in PBS solution, pH 7.2. Endotoxin levels were <0.5 EU/mL as determined by the Limulus amebocyte lysate coagulation assay (Limulus ES-II Test Wako, Wako Pure Chemical Industrials, Osaka, Japan). The purity and relative molecular weight of THP were detected by 10% SDS-PAGE under reducing conditions after staining with GelCode Coomassie blue solution (Figure 1A). The 80–90 kDa molecule was further identified as THP by anti-uromucoid antibody staining (The Binding Site Ltd, University of Birmingham Research Institute, Birmingham, UK), as described in our previous report [22].

### 3.3. Isolation of PMNs from Normal Human Peripheral Blood

Heparinized venous blood obtained from normal individuals was mixed with a one-quarter volume of 2% dextran solution (molecular weight: 475,000 Da) and incubated at room temperature for 20 min. The leukocyte-enriched supernatant was collected and gently layered over Ficoll-Hypaque density gradient solution (specific gravity 1.077) followed by centrifugation at 300 ×*g* for 30 min. The PMNs were collected from the bottom. The residual RBCs in the PMN suspension were lysed in chilled 0.85% ammonium chloride solution. The viability and purity of the PMN preparation were detected by trypan blue dye exclusion and Wright’s stain, respectively. The cell concentration of PMNs was adjusted to 2 × 10^6^/mL in 10% fetal bovine serum (FBS) in RPMI-1640 medium (10% FBS-RPMI). All of the urine and blood donors provided informed consent, approved by the Internal Review Board and Medical Ethics Committee, National Taiwan University Hospital, Taipei, Taiwan.

### 3.4. Detection of PMN Phagocytosis by Flow Cytometry

Fluoresbrite carboxylate microspheres (diameter: 0.75 μM) were previously opsonized with fresh human serum by incubation at 37 °C for 2 h. Freshly prepared PMNs (2 × 10^6^ cells/mL) were pretreated with medium or with different protein kinase inhibitors including PD98059 (50 μM), SB203580 (1 μM), and wortmannin (0.1 μM) at 37 °C for 20 min, and then mixed with the opsonized beads (1 × 10^8^ beads/mL) in the presence of culture medium, LPS (20 ng/mL), THP (10 μg/mL) or human EGF (20 ng/mL) at 37 °C in 5% CO_2_/95% air for 60 min. After several washes with PBS, pH 7.2, the PMNs were fixed in 4% paraformaldehyde to stop phagocytosis. The percentage (%) and mean fluorescence intensity (MFI^#^, denoted by mean channel number) of PMN phagocytosis were determined by FACSort flow cytometry (Becton-Dickinson, Mountain View, CA, USA) with 488 nm excitation. We confirmed that the beads were phagocytosed but not attached to PMNs, because pretreatment of PMNs with the cytoskeleton inhibitors colchicine (2 μg/mL) or cytochalasin B (10 μg/mL) remarkably reduced neutrophil phagocytosis.

### 3.5. Preparation of Whole Cell Lysates

The human promyelocytic leukemia cell line HL-60, subcultured in Iscove’s modified Dulbecco’s medium containing 10% FBS, was stimulated with 2 μM all-trans retinoid acid in 95% air/5% CO_2_ at 37 °C for 5–7 days. The differentiated PMN-like HL-60 cells (2 × 10^6^ cells/mL), identified by morphological changes and by CD16 expression on the cell surface (detected by flow cytometry) were then incubated with LPS (20 ng/mL), THP (10 μg/mL), or human recombinant EGF (20 ng/mL) for different periods. The treated cells were centrifuged at 800 ×*g* for 5 min, followed by washing with cold PBS. The cell pellet was lysed with cold RIPA buffer (25 mM Tris-HCl, pH 7.6, 150 mM NaCl, 1% NP-40, 1% sodium deoxycholate, and 0.1% SDS) containing a protease inhibitor and phosphatase inhibitor cocktail (Roche, Mannheim, Germany) in an ice bath for 30 min. The cell lysates were centrifuged at 13,500 rpm at 4 °C for 15 min to remove debris. The protein concentration of each cell lysate was determined by the Bradford method. 

### 3.6. Western Immunoblotting

The cell lysates were separated by 12% SDS-PAGE and transferred to polyvinylidene fluoride membrane (PVDF; Millipore Inc., Billerica, MA, USA) in a Mini Trans-Blot cell (Bio-Rad, Hercules, CA, USA) for 2 h at 350 mA. The PVDF membranes were blocked with Tris-buffered saline and Tween 20 (TBST: 50 mM Tris, 150 mM NaCl, 0.05% Tween 20, pH 7.6) containing 1% BSA at room temperature for 30 min. The dispersed protein molecules were probed with a specific antibody against MAPK signaling molecules at 4 °C for 16 h. After several washes with TBST, the complexes were detected by HRP-conjugated goat anti-mouse Ig antibodies using an enhanced chemiluminescence protein detection system (Amersham International, Amersham, UK).

### 3.7. Statistical Analysis

The results were assessed by the non-parametric Wilcoxon’s signed-rank test to compare the difference between two groups. In addition, the Krushal-Wallis test was employed for multiple comparisons among different groups. A *p* < 0.05 was considered statistical significance.

## 4. Conclusions

At least four original findings are observed in the present study: (A) THP-enhanced PMN phagocytosis is p38 and ERK1/2 MAP kinase dependent; (B) THP activates Rho family small GTPase molecules Cdc42 and Rac in PMN; (C) The signaling pathways induced by THP and LPS differ somewhat in their kinetics and strength; and (D) EGF enhances PMN phagocytosis additive with LPS, but not with THP. We are the first to find that THP activates the p38 and ERK1/2 MAPK signaling pathways via EGFR-dependent mechanism to augment PMN phagocytosis. 
